# The Impact of Implementation of Bundle to Reduce Catheter-Related Bloodstream Infection Rates

**DOI:** 10.14740/jocmr2314w

**Published:** 2015-09-25

**Authors:** Mayra Goncalves Menegueti, Kym Marcel Martins Ardison, Fernando Bellissimo-Rodrigues, Gilberto Gambero Gaspar, Olindo Assis Martins-Filho, Marcelo Lourencini Puga, Ana Maria Laus, Anibal Basile-Filho, Maria Auxiliadora-Martins

**Affiliations:** a Division of Intensive Care, Department of Surgery and Anatomy, Ribeirao Preto Medical School, University of Sao Paulo, SP 14049-900 Ribeirao Preto, Brazil; b Getulio Vargas Foundation, Brazilian School of Economy and Finance, Rio de Janeiro, Brazil; c Department of Social Medicine, Ribeirao Preto Medical School, University of Sao Paulo, SP 14049-900 Ribeirao Preto, Brazil; d Hospital Infection Control Committee, Ribeirao Preto Medical School, University of Sao Paulo, SP 14049-900 Ribeirao Preto, Brazil; e Laboratorio Laboratory of Biomarkers, Rene Rachou Institute, Oswaldo Cruz Foundation, Belo Horizonte, Minas Gerais, Brazil; f Ribeirao Preto Nursing School, University of Sao Paulo, SP 14049-900 Ribeirao Preto, Brazil

**Keywords:** Intensive care unit, Catheter-related bloodstream infection, Central venous catheter, Educational intervention

## Abstract

**Background:**

The aim of the study was to investigate how control bundles reduce the rate of central venous catheter-associated bloodstream infections (CVC-BSIs) rates in critically ill patients.

**Methods:**

This is a prospective before-and-after study designed to evaluate whether a set of control measures (bundle) can help prevent CVC-BSI. The bundles included a checklist that aimed to correct practices related to CVC insertion, manipulation, and maintenance based on guidelines of the Center for Disease Control and Prevention (CDC).

**Results:**

We examined 123 checklists before and 155 checklists after implementation of the training program. Compared with the pre-intervention period, CVC-BSI rates decreased. Hand hygiene techniques were used correctly. CVC-BSI incidence was 9.3 and 5.1 per 1,000 catheter-days before and after the training program, respectively.

**Conclusions:**

The implementation of a bundle and training program effectively reduces CVC-BSI rates.

## Introduction

Hospital care has been associated with hospital-acquired infections (HAIs). Even though HAIs are almost always preventable, they constitute an important source of morbidity and mortality. Intensive care units (ICUs) assist critically ill patients by means of invasive techniques, which expose patients to hospital microorganisms and potential infection [1]. Central venous catheters (CVCs) are widely employed in hospital environments, especially in the ICU, to monitor patients and administer medications. In Europe, the main cause of HAI is CVC-related primary bloodstream infection (CVC-BSI) [2].

The incidence of CVC-BSI in ICUs in the USA varies between 2.9 and 11.3 per 1,000 catheters-day; mortality lies between 12 and 25% [3]. In the study “The Evaluation of Processes and Indicators in Infection Control (EPIC)” involving 10,038 patients in 1,417 ICUs in Europe, CVC-BSI accounted for 12% of hospital infections [4].

In Brazil, the epidemiological surveillance center (ESC) recently published infection rates on the basis of notifications received from 341 hospitals. Laboratorial examinations confirmed CVC-BSI rates of 4.62 and 15.18 per 1,000 catheters-day in 50th and 90th percentile, respectively [5].

From the approximately 99,000 infection-related deaths per year, CVC-BSI causes an estimated 31,000 deaths, which cost an average of US$18,000 per infection [6, 7]. These infections extend the length of ICU and hospital stay up to 6 and 21 days, respectively. Mortality attributed to these infections is approximately 35%; costs rise by about US$56,000 per infection [8]. Hence, reducing the risk of HAI should decrease hospital stay duration and costs [9].

Epidemiological surveillance and a set of measures concerning CVC insertion and maintenance may effectively reduce the incidence of these infections [3]. A review paper listed the strategies that could diminish catheter colonization: insertion of the CVC into the subclavian vein instead of femoral or jugular sites, cutaneous antisepsis prior to CVC insertion, and training programs for the health staff followed by assessment of professional performance [9].

In 2002, Centers for Disease Control and Prevention (CDC) listed updated guidelines to prevent CVC-BSI. According to the recommendations, it is necessary to: 1) continuously train and develop the health team and conduct daily routine surveillance of the insertion site during dressing changes; 2) disinfect the hands before puncturing the access; 3) use elements of maximal sterile barrier (cap, mask, gowns, gloves, and goggles); 4) disinfect the insertion site and ensure antisepsis of a large field; 5) remove the catheter upon suspicion of local skin infection, catheter colonization, or CVC-BSI; 6) consider whether it is really essential to maintain a central catheter; and 7) avoid changing the central access on a routine basis [10]. The Institute for Healthcare Improvement (IHI) also recommended five measures to prevent CVC-BSI: 1) hand hygiene; 2) maximal sterile barrier during catheter insertion; 3) cutaneous antisepsis with chlorhexidine; 4) use of the femoral vein as central venous access only as a last resort in adult patients; and 5) daily assessment as to whether the central catheter is really necessary [11].

Data collected at our institution (1999 - 2009) revealed a CVC-BSI incidence of 11.3 per 1,000 catheters-day (unpublished data). This high infection rate, much higher than the 50th percentile in the state of Sao Paulo, has demanded that could improve infection control. In this context, this study aimed to assess whether the implementation of preventive bundles reduces CVC-BSI rates among critically ill patients.

## Methods

This quasi-experimental, before-and-after quantitative study was conducted in the ICU (total of nine beds) of a tertiary university hospital belonging to a government university. The Ethics Committee of Hospital das Clinicas da Faculdade de Medicina de Ribeirao Preto, University of Sao Paulo, Brazil (protocol 7076-2010) approved this study.

### Participants

All the patients admitted to the ICU and for whom CVC insertion was recommended were considered eligible for the study.

### Intervention

A multidisciplinary team designed the training program, which highlighted the correct techniques to insert, manipulate, and maintain the CVCs. The set of measures included specific recommendations for CVC insertion and maintenance, more specifically: 1) educating and training the staff responsible for catheter insertion and manipulation; 2) applying a checklist for each CVC insertion, such as the use of maximal sterile barriers during CVC insertion (cap, mask, sterile gown, sterile gloves, and large sterile sheet), hand hygiene, insertion site disinfection and antisepsis, choice of catheter insertion site, and number of puncture attempts; 3) substituting catheters as fast as possible when asepsis cannot be ensured; 4) avoiding regular replacement of catheters; 5) adopting a puncture kit to obtain central access; 6) changing venous access dressings that are wet, loose, or dirty - that is, every 2 and 7 days in the case of gauze and transparent bandages, respectively - and observing the presence of phlogistic signs; and 7) reassessing the need for the central access on a daily basis [10].

From 2008, every 6 months, resident doctors as well as the medical and nursing staff were trained on how to insert and maintain catheters. Routine visits were accomplished twice a week; bundles for CVC-BSI control were reinforced, the state of the central venous access with respect to the dressing was verified, and the need to maintain the device in place was assessed. The checklists filled in before and after staff training were compared, to verify whether the preventive bundles were being followed.

The CVC-BSI rates were registered from 1999 to 2007 (pre-intervention period) and from 2008 to 2011 (intervention period). Two hemoculture samples were collected for all the patients with suspected CVC-BSI; the samples were incubated for 5 - 7 days. To notify infection, nursing staff members participating in the program for hospital infection control examined the hemocultures on a daily basis. The isolated microorganism was classified as a contaminant that originated either from community-acquired or nosocomial infection according to the CDC criteria.

The infection rates were calculated using catheter-day as denominator. All the CVC-related infections were included.

### Outcome measures

Throughout the study, data on the number of catheter-related BSIs and catheter-days were collected. CVC-BSI in adults is defined by the National Nosocomial Infections Surveillance System (NNISS) [10]. The definition of a central catheter is a catheter that ends at or near the heart or in a great vessel close to the heart. In accordance with the NNISS guidelines, we count the use of multiple lines in one patient as 1 catheter-day. We considered it to be CVC-BSI from an ICU if it was detected at least 48 h after admission to less than 48 h after discharge. CVC-BSI includes laboratory confirmed BSI and clinical sepsis. Laboratory confirmed BSI must meet one of the following criteria. Criteria 1: recognized pathogen isolated from blood culture. Pathogen must not be related to infection at another site.” Criteria 2: one of the following: fever (38 °C), chills, or hypotension and any of the following: 1) common skin contaminant isolated from two blood cultures drawn on separate occasions and organism must not be related to infection at another site; 2) common skin contaminant isolated from blood culture from patient with intravascular access device and physician institutes appropriate antimicrobial therapy; 3) positive antigen test on blood and organism must not be related to infection at another site.

Clinical sepsis must meet either of the following criteria: patient has at least one of the following clinical signs with no other recognized cause: fever (> 38 °C), hypotension (systolic pressure < 90 mm Hg), or oliguria (< 20 mL/h), and blood culture not done or no organisms or antigen detected in blood and no apparent infection at another site, and physician institutes treatment for sepsis [10].

### Statistical analysis

The data were collected and entered in the database of the software SPSS (SPSS Inc., Chicago, IL, USA). The mean values of CVC-BSI incidence density were represented graphically; infection rates during the pre-intervention and intervention periods were compared by multiple linear regression of time series and the values were expressed as percentage.

## Results

We verified a total of 123 and 155 checklists filled in before and after the start of the training program. The professionals corrected the technique they used to conduct hand hygiene. Prior to the training, 71% of the staff members disinfected the hands with the antiseptic chlorhexidine, 11% disinfected the hands with alcoholic solution, 7% disinfected the hands with a combination of chlorhexidine and alcoholic solution, and 7% used an incorrect technique. Hand hygiene was not verified for 4% of the professionals. Following the training, 75% of the staff used chlorhexidine, 9% used alcoholic solution, and 14% used a combination of chlorhexidine and alcoholic solution for hand disinfection. Hand hygiene was not verified for 2% of the staff members. Table 1 describes the results obtained for all the measures.

**Table 1:** Percent of Compliance With CVC-BSI Preventive Bundles in the Pre- and Post-Training Periods, Based on a Check-List Employed in the ICU

Measure	Before training	After training
Personal protection equipment (PPE)		
Protection goggles	37%	71%
Cap, mask, sterile gown and sterile clothes	93%	100%
Disinfection and antisepsis of the catheter insertion site	90%	100%
Catheter insertion site		
Subclavian vein	33%	55%
Jugular vein	42%	36%
Femoral vein	25%	9%
Use of sterile fields		
Large fields	64%	100%
Partial coverage (insertion site)	36%	-
Puncture attempts		
1	44%	57%
2	15%	16%
3 or more	41%	27%

**Figure 1:**
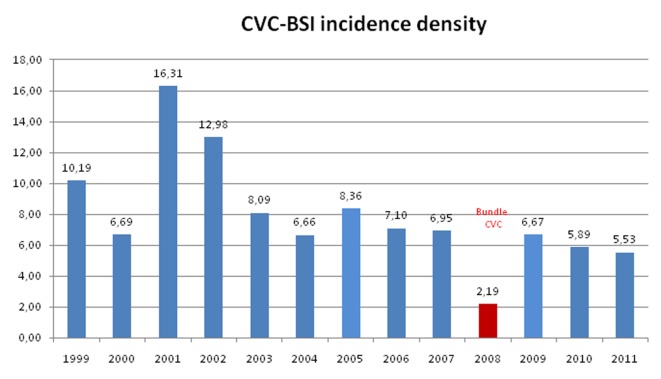
Annual mean CVC-BSI incidence density in the periods before and after implementation of the training program and preventive bundles.

Comparison of the mean values of CVC-BSI incidence density showed that the difference between the means dropped considerably, especially during the first intervention year (Fig. 1). The set of measures implemented from 2008 evidenced lower CVC-BSI rates as compared with the pre-intervention period. The mean CVC-BSI incidence density before and after implementation was 9.3 and 5.1 cases per 1,000 catheters-day, respectively.

After implementation of the bundles, the mean reduction in CVC-BSI episodes was 4.3 per 1,000 catheters-day (95% CI: 2.27 - 6.35) (Table 2). Therefore, assuming a significance level of 5%, it was evident that the mean CVC-BSI incidence fell during the period of program implementation.

**Table 2:** Multiple Linear Regression Applied to the CVC-BSI Incidence Densities in the Periods Before and After Implementation of the Training Program and Preventive Measures

Variable	Coefficient	Confidence interval
Before intervention	9.226993	7.93 - 11.52
After intervention	-4.310572	-2.27-(-6.35)
Time trend	0.88493	0.098019

Data in Table 2 corroborated the positive effects of implementing the preventive bundles: CVC-BSI decreased along time. The coefficient for the variable time trend confirmed this observation. Basically, a coefficient lower than 1 corresponded to lower incidence during the post-implementation period (0.8 or 20% lower) as compared with the pre-training period.

## Discussion

Literature studies have shown that implementing preventive and educational measures impacts CVC-BSI rates positively [12].

Warren et al reported that training health professionals to prevent CVC-BSI reduced infections from 9.4 to 5.5 per 1,000 catheters-day [13]. Other studies have also described significantly lower CVC-BSI rates after implementation of control bundles. An investigation conducted in an ICU in the United Kingdom described 3.4 and no infection episodes per 1,000 catheters-day prior to and 19 months after adoption of preventive bundles, respectively [14].

The present study implemented a training program with bundles that aimed at CVC-BSI prevention, a routine practice of the local Hospital Infection Committee. In other words, this study approached a real-life issue and demonstrated that this type of intervention was positive: a set of inexpensive measures, such as training and adoption of a checklist, decreased the number of infections. Lobo et al also verified lower infection rates after implementing an educational program: from 20 to 11 per 1,000 catheters-day within 16 months, a reduction of approximately 40% [15].

However, when the CVC-BSI rate was compared year to year in this study, the rate was quite high in the years of the pre-education period. It then exhibited a decrease no primer year post intervention, but they rebounded in the year following the intervention, suggesting that training was no effective and should be made permanent.

Yilmaz et al also reported that in the post education period, the rate of CVC-BSI was low in the first 3 months but rose by 2.08 times from the third month onward. Consequently, a regular educational program focusing on the nurses, interns, residents and doctors responsible for catheter insertion and maintenance will markedly reduce CVC-BSI rates. A regular in-service educational program that includes the most up-to-date developments in the field, the monthly publication of CVC-BSI rates, and rewards for positive results will prevent undesirable results in terms of morbidity, mortality, and cost [16].

This study was fundamental to find out whether a continued education program would impact the Brazilian ICU routine, as verified in studies conducted in the USA and Europe. Comparison of international infection rates did reveal different data among countries. However, the present study evidenced that the set of measures to prevent CVC-BSI were also effective in Brazil, despite the distinct scenario.

The present study presents some limitations. 1) We only monitored the adopted bundles during visits to the ICU and not along all the periods, on a daily basis. Nevertheless, the bundles were effective: infection rates diminished without applying any other significant intervention during the period. 2) We were not able to identify the impact associated with each of the implemented measures. On the other hand, because we adopted low-cost, easy-to-implement bundles, this limitation became less relevant. 3) This study included one center only.

### Conclusion

Our results demonstrated the importance of implementation and application of a set of measures and staffs training in reduce CVC-BSI rates. Consequently, a regular continuing education program focusing on the nurses, interns, residents and doctors responsible for catheter insertion and maintenance is mandatory and will markedly reduce CVC-BSI rates.

## Author Note

This study was conducted at the adult Intensive Care Unit of the Clinics Hospital of Ribeirao Preto Medical School, University of Sao Paulo, Brazil.
